# Origin and Evolution of Plant Long Terminal Repeat Retrotransposons with Additional Ribonuclease H

**DOI:** 10.1093/gbe/evad161

**Published:** 2023-09-11

**Authors:** Mikhail Biryukov, Kirill Ustyantsev

**Affiliations:** Sector of Molecular and Genetic Mechanisms of Regeneration, Institute of Cytology and Genetics SB RAS, Novosibirsk, Russia; Novosibirsk State University, Novosibirsk, Russia; Sector of Molecular and Genetic Mechanisms of Regeneration, Institute of Cytology and Genetics SB RAS, Novosibirsk, Russia

**Keywords:** LTR retrotransposons, retroviruses, ribonuclease H, plants, convergent evolution

## Abstract

Retroviruses originated from long terminal repeat retrotransposons (LTR-RTs) through several structural adaptations. One such modification was the arrangement of an additional ribonuclease H (aRH) domain next to native RH, followed by degradation and subfunctionalization of the latter. We previously showed that this retrovirus-like structure independently evolved in *Tat* LTR-RTs in flowering plants, proposing its origin from sequential rearrangements of ancestral *Tat* structures identified in lycophytes and conifers. However, most nonflowering plant genome assemblies were not available at that time, therefore masking the history of aRH acquisition by *Tat* and challenging our hypothesis. Here, we revisited *Tat's* evolution scenario upon the aRH acquisition by covering most of the extant plant phyla. We show that *Tat* evolved and obtained aRH in an ancestor of land plants. Importantly, we found the retrovirus-like structure in clubmosses, hornworts, ferns, and gymnosperms, suggesting its ancient origin, broad propagation, and yet-to-be-understood benefit for the LTR-RTs’ adaptation.

**Significance**

Previously, we showed that a hallmark of animal retroviruses’ evolution—an acquisition of an additional ribonuclease H domain followed by degradation of the native one—independently evolved in long terminal repeat retrotransposons (LTR-RTs) of the *Tat* lineage from flowering plants. However, poor phylogenetic diversity of nonflowering plant genomes available at the time hindered the history of the domain acquisition and early evolution of the *Tat* lineage. Here, we redate the origin of *Tat* LTR-RTs to the common ancestor of liverworts, mosses, and hornworts, expand the diversity of known *Tat* structures, and show the presence of the retrovirus-like “dual ribonuclease H” structure in most extant plant phyla, indicating its important, yet not fully understood, function for the LTR-RTs’ adaptation.

## Introduction

Evolutionary studies suggest that retroviruses most probably originated from a common ancestor with Ty3/gypsy long terminal repeat retrotransposons (LTR-RTs). Indeed, they share a general order and content of the encoding protein domains in the polyprotein (Pol) with Ty3/gypsy LTR-RTs, and the intracellular reverse transcription–mediated replication cycle as well as the genome integration mechanism with most extant LTR-RTs (Xiong and Eickbush 1990; Havecker et al. 2004; Basu et al. 2008; Eickbush and Jamburuthugoda 2008). A hallmark of retroviruses’ evolution from LTR-RTs was the acquisition of a third open reading frame (ORF)—the envelope (*env*) gene. Env is the key protein mediating horizontal transmission and the infectious ability of retroviruses (Malik et al. 2000; Havecker et al. 2004). Divergent *env*-like ORFs of independent origin were identified in some LTR-RTs lineages from insects, nematodes, and plants (Malik et al. 2000; Wright and Voytas 2002). However, Wang and Han (2022) recently discovered a group of *env*-less LTR-RTs in sea anemones, named *Odin*, which together with lokiretroviruses (Wang and Han 2021) form a sister lineage to all known vertebrate retroviruses (Wang and Han 2022). *Odin* LTR-RTs highlight another feature of evolution from LTR-RTs to retroviruses: acquisition of an additional ribonuclease H (aRH) domain downstream of native RH in the *pol* gene. In this dual RH assembly, the native RH domain is degenerated into a tether domain, lacking its catalytic activity, but still retaining the canonical RH-like fold and substrate binding capacity (Malik and Eickbush 2001; Lapkouski et al. 2013; Wang and Han 2022). It was proposed that the tether RH domain fine-tunes the catalytic activity of aRH during the reverse transcription stage of the retroviral replication cycle (Basu et al. 2008; Delviks-Frankenberry et al. 2008; Lapkouski et al. 2013).

Previously, we described that retrovirus-like dual RH structure independently evolved in the Ty3/gypsy LTR-RTs of clade *Tat* in plants (Viridiplantae) and the *Chronos* and *Archon* LTR-RTs clades from parasitic protist oomycetes (Ustyantsev et al. 2015, 2017). However, the acquired aRH domain of *Tat*, *Chronos,* and *Archon*, phylogenetically belongs to the Archaea-like clade of RH type I genes, while aRH of *Odin* and retroviruses comes from the Fungi/Metazoa-like cluster (Wang and Han 2021, 2022). Interestingly, in all the LTR-RT lineages, we observed a typical degradation of the native RH domain while the aRH domain has a full set of the RH active center core amino acids DEDD (Ustyantsev et al. 2015, 2017). The recurrent emergence of the dual RH structure in LTR-RTs indicates its potential, yet to be understood, adaptive function and, thus, is important for understanding the early evolution of retroviruses toward their infectious state. One possible advantage of dual RH may be in putative increase of LTR-RTs genetic variability due to higher frequency of strand transfers during reverse transcription that comes from enhanced catalytic activity of aRH and more flexible RT-tether-aRH protein structure (discussed in Ustyantsev et al. 2015).

Among the abovementioned LTR-RTs, only in *Tat* we found multiple alternative structural variants to dual RH, differing by the aRH position relative to other retrotransposon domains. There we proposed the hypothesis that the retrovirus-like dual RH structure evolved in *Tat* LTR-RTs from flowering plants through sequential rearrangements of ancestral *Tat* structures, which emerged since the origin of lycophytes (Ustyantsev et al. 2015). However, this study was performed in 2014, and at that time, there were a limited number of genome assemblies from nonflowering plant taxa available, which can raise questions about the proposed evolution scenario. Encouraged by the fact that *Tat* LTR-RTs present a unique example to trace potential intermediate stages of the evolution of retroviruses in connection with the aRH acquisition, we sought to reanalyze the diversity of *Tat* LTR-RTs in the light of the currently available genome assemblies of nonflowering plants. We broadened sampling of nonangiosperm genomes, now covering most of the major plant phyla. Using a recently developed sensitive algorithm for retrotransposons’ identification (Biryukov and Ustyantsev 2022), we re-evaluated the origin, diversity, and distribution of aRH-containing LTR-RTs in green plants. Based on this, we propose a new scenario for *Tat* LTR-RTs evolution upon the aRH domain acquisition.

## Results and Discussion

### Origin and Diversity of *Tat* LTR-RTs

**Fig. 1.**
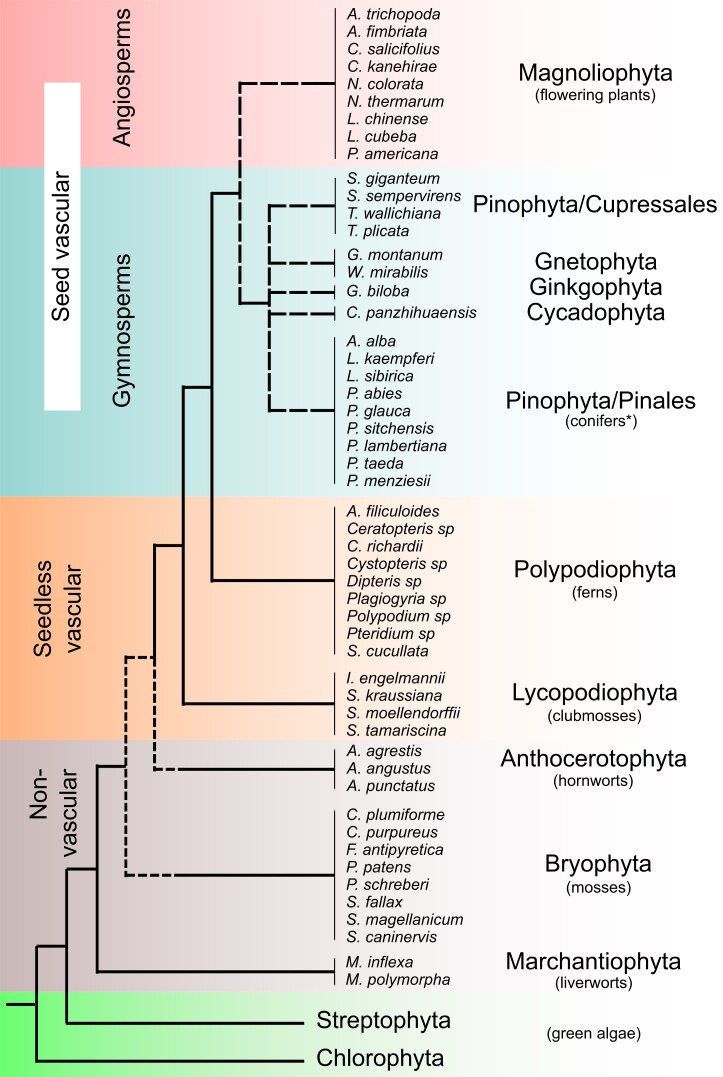
A schematic phylogenetic tree of green plants and studied land plant taxa. See supplementary table S1, Supplementary Material online for more details on the taxonomy and studied species. The phylogenetic tree as in Leebens-Mack et al. (2019). Dashed branches indicate not fully resolved topologies. Common names of the phyla used throughout the study are in parentheses under the phyla names. The common name “conifers,” which is sometimes used to designated gymnosperms in general, is assigned only to Pinaceae family in this study. Branches of green algae (Streptophyta and Chlorophyta) are collapsed as no aRH-containing LTR-RTs were identified in their genomes.

**Fig. 2.**
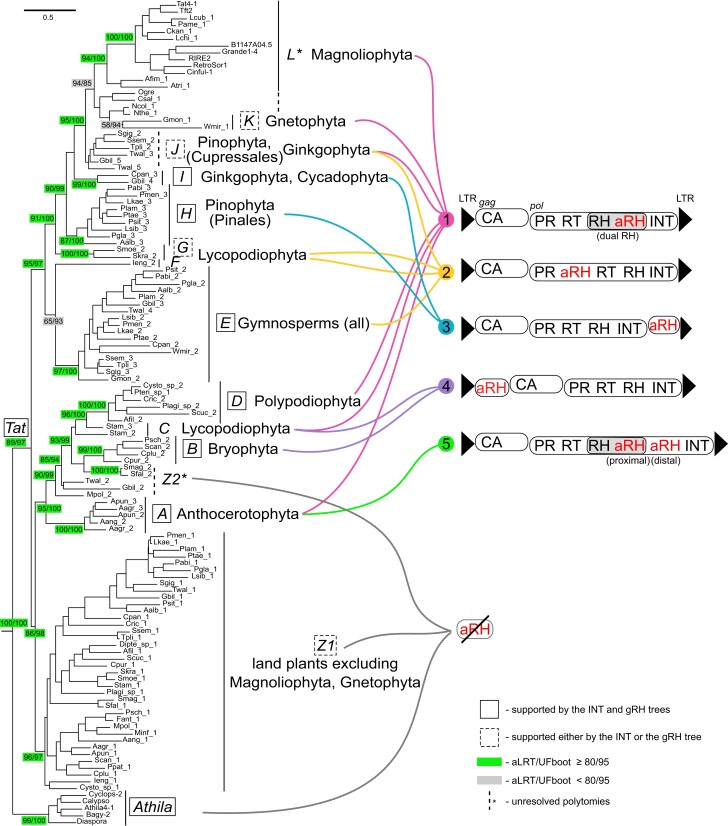
Structural diversity and phylogenetic relationships among *Tat* LTR-RTs. Variability of identified structures of *Tat* LTR-RTs is shown on the right. Structures are enumerated from the highest frequency of occurrence to the lowest. Ovals, ORFs. Capsid domain of the Gag protein, CA. The maximum-likelihood phylogenetic tree was reconstructed based on amino acid sequences of the RT domain from representatives of *Tat*, *Athila*, and other Ty3/gypsy lineages (outgroup, not shown). Capital italicized letters (*A–L*, *Z1*, and *Z2*) designate *Tat* clusters with distinct structural and/or taxonomic representatives. See figure 1 and supplementary tables S1 and S3, Supplementary Material online for complete taxonomic information and additional details on each *Tat* LTR-RT representative. See supplementary figures S1 and S2, Supplementary Material online for comparison with the concatenated RT and INT and separate INT and gRH phylogenies, respectively. aLRT/UFboot branch support values are shown only for major selected clusters/nodes.

Recently, we developed an algorithm for Domain-Associated RetroTransposon Search (DARTS) (Biryukov and Ustyantsev 2022). We showed that DARTS is up to eight times more sensitive than de novo LTRharvest-based implementation used to mine aRH-containing LTR-RTs from the genomes of green plants in our previous study (Ellinghaus et al. 2008; Biryukov and Ustyantsev 2022). Here, using DARTS, we analyzed genomic sequences of 92 species of green plants spanning most of the major phyla (figs. 1 and 2; supplementary table S1, Supplementary Material online). Altogether, in 43 genomes, we identified five structural variants (#1–#5) of LTR-RTs with aRH, which, based on the reverse transcriptase (RT) domain phylogeny, are distributed among 12 mostly taxon-specific clusters and denoted in Latin letters from *A* to *L* (figs. 1 and 2; supplementary tables S2 and S3, Supplementary Material online). All LTR-RTs with aRH clustered to the monophyletic *Tat* lineage (fig. 2), and the overall phylogeny of *Tat* followed vertical evolution of Viridiplantae (figs. 1 and 2; supplementary figs. S1 and S2, Supplementary Material online). Additionally, within *Tat* from all major land plant taxa, excluding flowering plants (Magnoliophyta) and Gnetophyta, we found two lineages lacking aRH—*Tat Z1* and *Z2* (fig. 2; supplementary figs. S1 and S2 and table S2, Supplementary Material online). The *Tat* lineages are also subgrouped into two major branches: 1) *Tat A–D*, *Z1* and *Z2* and 2) *Tat E–L.* Therefore, clusters *Z1* and *Z2* are not basal to all aRH-containing LTR-RTs and may exemplify cases of secondary loss of the aRH domain, if it was acquired by an ancestor of all *Tat* before the split into *Tat A–D*, *Z1* and *Z2*, and *Tat E–L*. Importantly, coclustering of *Tat Z1* and *Z2* with *Tat A–D* is also supported by the integrase (INT) phylogenetic tree (supplementary fig. S2, Supplementary Material online). The phylogenetic tree derived from concatenated RT and INT sequences supports the clusters identified on the RT phylogeny (supplementary fig. S1, Supplementary Material online).

We did not find any LTR-RTs containing aRH in Chlorophyta and Streptophyta algae. Overall, 40 species from these taxa were covered compared with four in the previous study (Ustyantsev et al. 2015), confirming that the aRH acquisition did not happen before the emergence of land plants. Interestingly, no LTR-RTs from the *Tat/Athila* clade were found in the algae genomes either, suggesting the acquisition of aRH and origin of the *Tat* lineage in land plants. Although no traces of aRH-containing LTR-RTs were detected in two liverwort species (Marchantiophyta), the earliest branching of the studied nonseed land plants (figs. 1 and 2; supplementary table S1, Supplementary Material online), in both *Marchantia* genomes, we found members of the *Tat Z1* and *Tat Z2* clades, indicating emergence of *Tat* before the split of liverworts (fig. 2; supplementary table S2, Supplementary Material online). aRH-containing *Tat* LTR-RTs appear in all the rest studied taxa except for four species of mosses (Bryophyta) and three species of ferns (Polypodiopsida) (fig. 2; supplementary table S2, Supplementary Material online). In hornworts (Anthocerotophyta, cluster *Tat A*), we found the retrovirus-like dual RH structure (structure #1) as well as a novel structure with two sequential aRH domains—proximal and distal to the native RH domain (structure #5) (fig. 2). Overall, structure #1 *Tat* LTR-RTs were found in the majority of the studied land plant taxa, excluding mosses (Bryophyta) and two gymnosperm taxa—conifers (Pinophyta and Pinales) and *Cycas panzhihuaensis* (Cycadophyta). In four mosses (*Tat B*) and one clubmoss (*Selaginella tamariscina*, Lycopodiophyta, *Tat C*), we identified *Tat* LTR-RTs with aRH positioned in a separate ORF upstream of *gag* (structure #4). Another structure with aRH in a separate ORF, upstream of *pol* (structure #3), was found in conifers (*Tat H*), *C. panzhihuaensis* (*Tat I*), and *Ginkgo biloba* (Ginkgophyta, *Tat I*). However, in *G. biloba*, structure #3 accounts for less than 4% of all identified *Tat* LTR-RTs; in Pinales, it comprises 20% of the elements on average, while in *C. panzhihuaensis*, it reaches the highest, 68% (supplementary table S2, Supplementary Material online). The second most frequently met *Tat* domain arrangement, with aRH positioned upstream of the RT domain, was structure #2 (fig. 2). We found structure #2 in three species of clubmosses (*Tat F* and *G*) and in all studied gymnosperm genomes (*Tat E* and *J*). In gymnosperms, structure #2 *Tat* LTR-RTs were the most abundant, excluding two Gnetophyta species (*Gnetum montanum* and *Welwitschia mirabilis*) with dominating retrovirus-like structure #1 (∼90% of all elements) (supplementary table S2, Supplementary Material online). Finally, only structure #1 was detected in early branching angiosperms (Magnoliophyta, *Tat L*) and in ferns (Polypodiophyta, *Tat D*) (fig. 2).

### Unclear Phylogeny and Acquisition Scenarios of the aRH Domain

Previously, we showed that the aRH domain in *Tat* LTR-RTs clustered together with the RH genes from Archaea, green plants, some bacteria, and diverse nonfungi/nonmetazoan eukaryotes in the so-called archaeal ribonuclease H clade (Smyshlyaev et al. 2013; Ustyantsev et al. 2017). Similarly, all the aRH domains we found in *Tat* LTR-RTs positioned in the archaeal RH clade, while the native RH domains clustered separately with the RH domains of other LTR-RTs (supplementary fig. S3, Supplementary Material online). An overall phylogeny of the *Tat* LTR-RTs aRH domains did not follow the ones of the RT, INT, and native RH domains and had low resolution (fig. 2; supplementary figs. S2 and S3, Supplementary Material online). There was not enough statistical support for most of the clusters, and we must acknowledge that the deep-branched phylogeny of aRH remains unresolved. Potentially, this may be explained by the short length of the domain (∼130 amino acids), fast evolutionary rate of LTR-RTs, and varying divergence rates of aRH in different *Tat* structures. Therefore, we could equally assume that aRH was acquired once in the evolution of the *Tat* lineage or that there were multiple independent captures from either a single source or multiple sources. However, the scenario of a single acquisition from an ancestral host plant genome with subsequent interelement recombination at different evolutionary time points seems more plausible and parsimonious. Alternatively, we would need to assume that a repetitive number of low-probability events happened in a specific lineage of plant LTR-RTs and only there.

### Tether-Like Degradation of the Native RH Domain and Structural Evolution of *Tat* LTR-RTs

**Fig. 3.**
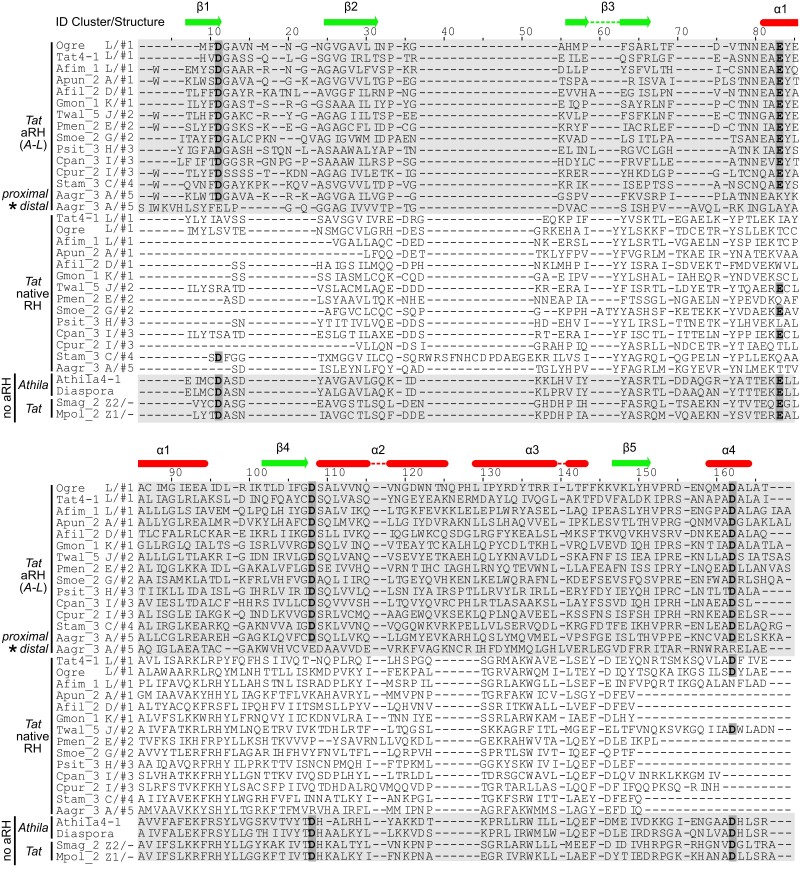
Tether-like catalytic center degradation in native *Tat* RH is invariant of the aRH position in the Pol domain assembly. Multiple amino acid sequence alignments of various *Tat* LTR-RTs’ aRH, corresponding native RH domains, and *Athila* RH are shown. Conserved DEDD catalytic core amino acids are highlighted in bold. *Tat* LTR-RTs structures (#1-#5), clusters (*A*–*L*, *Z1*, and *Z2*), and element names are as in figure 2 and supplementary table S3, Supplementary Material online. Schematics of the RH domain secondary structure are shown at the top of the image. An asterisk to the left of the Aagr_3 A/#5 distal aRH domain indicates degradation of the catalytic core amino acids relative to its intact proximal aRH.

In previous studies, we showed that acquisition of aRH in a retrovirus-like position (structure #1) caused tether-like degradation of the native RH domain in *Tat*, *Chronos*, and *Archon* LTR-RTs (Ustyantsev et al. 2015, 2017). Here, we found that native RH underwent degradation in all identified *Tat* structures with aRH, regardless of the aRH domain position (fig. 3). At the same time, we did not find the degeneration of native RH in the *Tat Z1* and *Z2* lineages without aRH, suggesting that they lost the aRH domain before the degradation had happened in other *Tat* lineages. An alternative, less parsimonious, scenario would assume that ancestors of *Tat Z1* and/or *Tat Z2* had never had aRH, requiring at least two independent aRH acquisitions by ancestors of the *Tat A–D* and *Tat E–L* lineages. However, in contrast to *Tat Z1*, *Tat Z2* cocluster within *Tat A–D* on both RT and INT phylogenies (fig. 2; supplementary fig. S2, Supplementary Material online), supporting (at least in this lineage) the secondary aRH loss hypothesis as well as the aRH acquisition before diversification of liverworts. Interestingly, we also observed degradation of the second, distal, aRH domain in *Tat* LTR-RTs from hornworts with structure #5, while the first, proximal, aRH retained most of its catalytic core amino acids (*Tat A*, figs. 1–3). This suggests that elimination of functional redundancy may be a primary reason for the domain degradation. Thus, changes in native RH should have occurred after the aRH acquisition by an ancestral *Tat* element. Although the original position of the acquisition remains enigmatic, the structure #1 position seems most plausible by analogy and frequency of occurrence. First, all the known examples of convergent dual RH emergence in LTR-RTs from animals, plants, and oomycetes show signs of the native RH domain degradation (Malik and Eickbush 2001; Ustyantsev et al. 2017; Wang and Han 2021, 2022). Second, the majority of the studied land plant taxa, including the earliest branching ones, have structure #1 (fig. 2). Structures #3 and #4 are rare and aRH is placed outside the *pol* gene raising questions on how the domain could function from a separate ORF, affecting the degradation of the native one. Structure #5 appears as a direct derivative of structure #1, probably, through duplication of the originally acquired aRH. Structure #2, on the other hand, is very frequent in gymnosperms, its aRH positioned within the *pol* ORF, suggesting it can be actively involved in the reverse transcription complex (Lapkouski et al. 2013; Nowak et al. 2014). Structure #2 might thus also have emerged at the beginning and caused the degeneration of native RH in an ancestral *Tat* LTR-RT element.

### An Updated Scenario for *Tat* LTR-RTs’ Evolution

**Fig. 4.**
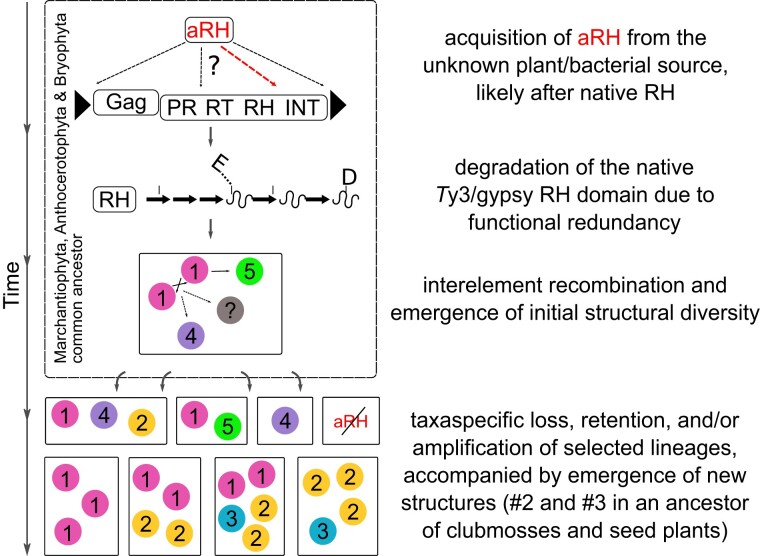
Proposed principal evolution of the *Tat* clade given a single time acquisition of aRH by an ancestral *Tat* element. *Tat* LTR-RT structures are depicted as circles with numbers as in figure 2. See figure 3 for details on degradation of the native RH domain. Hypothetical ancestral plant lineages are presented as square blocks.

Figure 4 summarizes our revised view on the emergence and evolution of *Tat* LTR-RTs. Apart from the obvious shift in the origin of aRH-containing LTR-RTs and the *Tat* lineage from an ancestor of vascular land plants to an ancestor of nonvascular land plants (figs. 1 and 2), we addressed that among the five identified structural variants, the dual RH structure emerged at the beginning of the *Tat* clade diversification. Other structures, probably, initially emerged from interelement recombination between structure #1 elements that happened early in the green plants’ evolution. Structure #4 emerged in a common ancestor of Bryophyta and Lycopodiophyta and structures #2 and #3 in an ancestor of the Lycopodiophyta and seed plants (fig. 2). The observed distribution of the structures among plant taxa could be best explained by selective taxa-specific loss and amplification of different *Tat* LTR-RT lineages rather than independent emergence. In the light of this new data, and through acknowledging insufficient resolution of the aRH phylogeny (supplementary fig. S3, Supplementary Material online), we reject our previous assumption (Ustyantsev et al. 2015) on several independent acquisitions of aRH by separate *Tat* lineages as nonlikely. Finally, we can conclude that the retrovirus-like dual RH arrangement ended up as the most persistent structure, suggesting its adaptive, yet remained to be understood, function for the LTR-RTs propagation in competition with other lineages throughout green plant and *Tat* LTR-RTs evolution.

## Materials and Methods

### Mining of LTR-RTs

The list of the studied genome assemblies, including the species taxonomy, is presented in supplementary table S1, Supplementary Material online. LTR-RTs with and without aRH as well as their conserved protein domains were mined using the previously developed DARTS v.01 algorithm (Biryukov and Ustyantsev 2022). The RPS-BLAST profiles and the software parameters were used as in the example from the original article (Biryukov and Ustyantsev 2022). DARTS was also used to identify LTRs, their length, and sequence identity; to reconstruct the overall domain structure of the LTR-RTs; and to precluster similar copies based on the RT domain amino acid sequence similarity and extract the clusters’ representatives as having the highest DARTS completeness score. Representatives with questionable or chimeric domain compositions were additionally manually checked.

### Phylogenetic Analysis

Amino acid sequences of the mined RT, INT, and native RH domains were aligned to corresponding profile alignments with the domains from known LTR-RT representatives from *Tat*, *Athila*, and other known Ty3/gypsy LTR-RT clades using MAFFT v. 7.505 (Katoh and Standley 2013); truncated and/or poorly aligned sequences were manually removed from the alignment. The RT, INT, and RH alignments were subjected to separate phylogenetic analyses. Draft approximate phylogenetic trees were built with FastTree v. 2.1.10 (Price et al. 2010) to check if the redundancy of LTR-RT representatives isolated from the same species in the same clusters could be further reduced, and, when applicable, the representatives with the highest DARTS completeness scores were retained for the final phylogenetic analysis. To avoid inclusion of potential sequencing artifacts, additional filtering was applied to select the final set of the representatives (supplementary table S3, Supplementary Material online). A lineage was removed if it met at least two of the following criteria: 1) does not cluster with lineages from closely related taxa which genomes were sequenced by the same institute on the RT or INT phylogenies; 2) has a unique predicted structure; 3) its sequence occupies a whole contig and/or lacks predicted LTRs.

For the aRH phylogenetic reconstruction, aRH and native RH amino acid sequences from the retained *Tat* LTR-RT representatives were aligned to a profile of diverse RH sequences from our previous study (Ustyantsev et al. 2017) using MAFFT. The final concatenated and RH phylogenetic trees were built using IQ-TREE v. 1.6.10 with the automatic estimation of the best substitution models (Minh et al. 2020). Ultrafast bootstrap (UFboot) and approximate likelihood ratio test (aLRT) were applied to estimate statistical support of the trees’ clustering (Anisimova and Gascuel 2006; Hoang et al. 2018). Phylogenetic clusters with support values above or equal to 80/95 (aLRT/UFboot) were considered credible according to the IQ-TREE manual.

### Comparison of Native RH and aRH Domains

Conservation/degradation analysis of the native RH and aRH domains coupled with their secondary structure prediction was performed as previously described (Ustyantsev et al. 2015, 2017).

## Supplementary Material

evad161_Supplementary_Data

## Data Availability

All data are available in the main text or Supplementary material online, as well as a public repository at GitHub: https://github.com/Mikkey-the-turtle/Supplementary_Data.
