# Supplementary material for: Origin and Evolution of Plant Long Terminal Repeat Retrotransposons with Additional Ribonuclease H
Source: Genome Biol Evol. 2023 Sep 11;15(9):evad161. doi: 10.1093/gbe/evad161 (PMC10508981; doi:10.1093/gbe/evad161)
Supplement: evad161_Supplementary_Data [file evad161_supplementary_data.pdf]

Supplementary Materials for

Origin and evolution of plant LTR retrotransposons with additional ribonuclease H

Mikhail Biryukov and Kirill Ustyantsev*

Sector of Molecular and Genetic Mechanisms of Regeneration, Institute of Cytology and Genetics SB RAS, 630090 Novosibirsk, Russia

*Corresponding author e-mail: ustyantsev@bionet.nsc.ru

This PDF file includes:

Figs. S1, S2, and S3
Tables S1, S2, and S3

Raw FASTA-formatted multiple sequence alignment data used to generate phylogenetic trees and sequences of the identified representative LTR retrotransposons and their conserved protein domains domains are available online at GitHub: https://github.com/Mikkey-the-turtle/Supplementary_Data

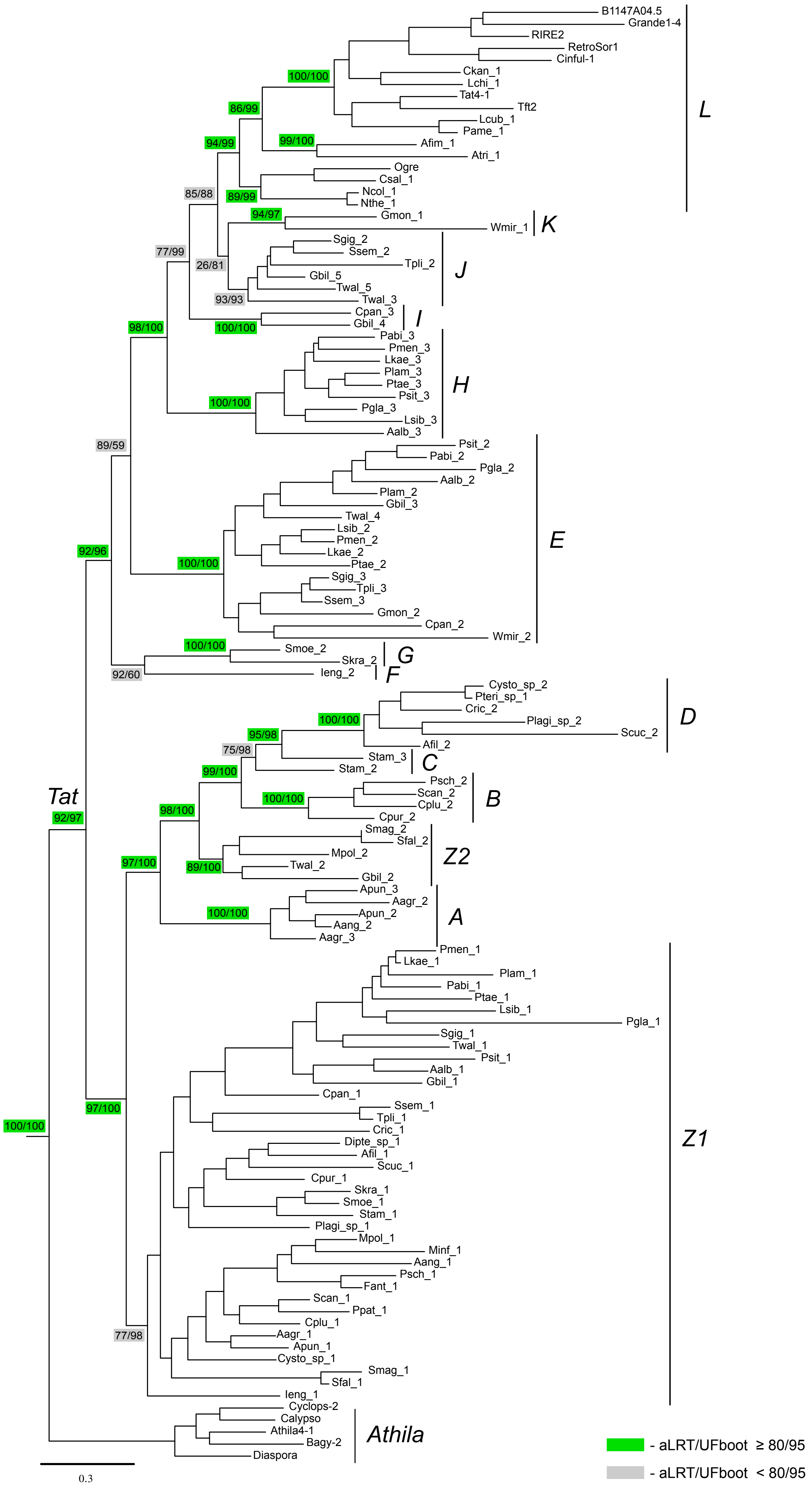

Figure S1. Phylogenetic relationships among *Tat* LTR-RTs. The maximum-likelihood phylogenetic trees were reconstructed based on concatenated amino acid sequences of the RT and INT domains from representatives of *Tat*, *Athila*, and other Ty3/gypsy lineages (outgroup, not shown). Capital italicized letters (A-L, Z1, Z2) designate *Tat* clusters with distinct structural and/or taxonomic representatives and correspond to those in Figure 2. See Supplementary Table 1 for complete taxonomic information and additional details on each *Tat* LTR-RTs representative. See Figure 2 to compare with the RT phylogeny. aLRT/UFboot branch support values are shown only for major clusters/nodes.

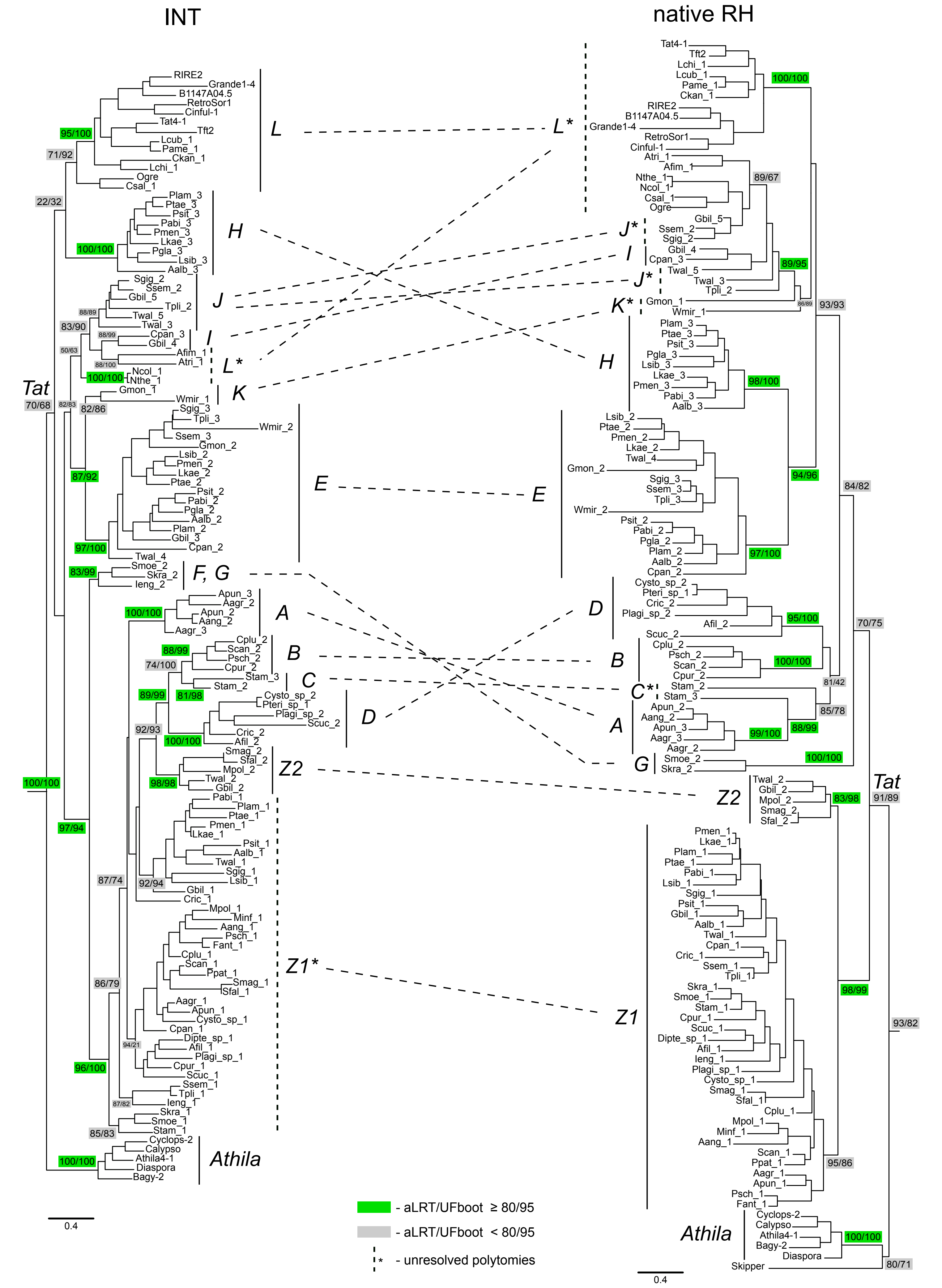

Figure S2. Phylogenetic relationships among *Tat* LTR-RTs. The maximum-likelihood phylogenetic trees were reconstructed based on amino acid sequences of the INT (left) and RH (right) domains from representatives of *Tat*, *Athila*, and other Ty3/gypsy lineages (outgroup, not shown). Capital italicized letters (A-L, Z1, Z2) designate *Tat* clusters with distinct structural and/or taxonomic representatives and correspond to those in Figure 2. See Supplementary Table 1 for complete taxonomic information and additional details on each *Tat* LTR-RTs representative. See Figure 2 and Supplementary Figure S1 to compare with the RT, and RT-INT phylogenies, respectively. aLRT/UFboot branch support values are shown only for major clusters/nodes.

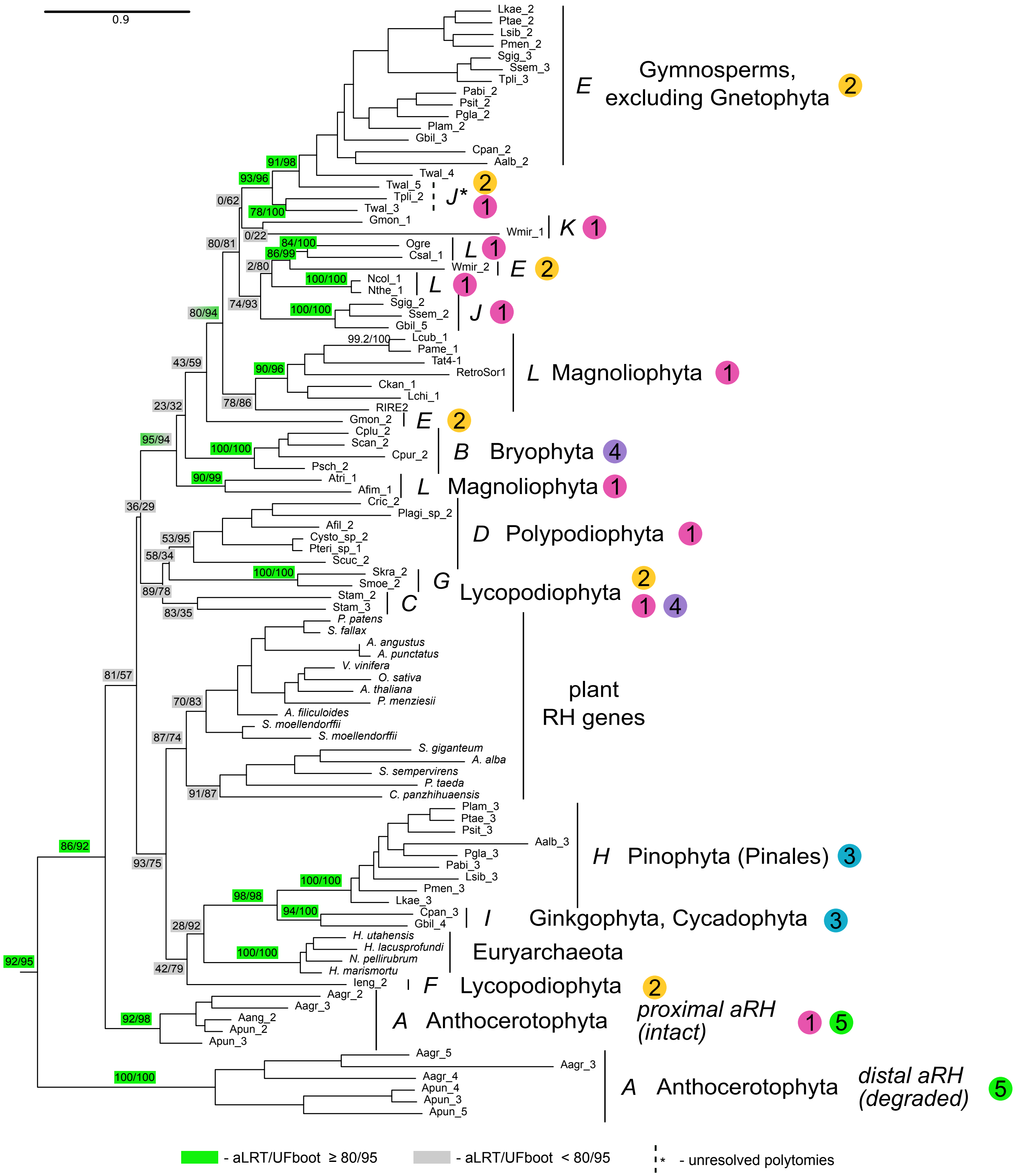

Figure S3. Phylogenetic relationships of the aRH domains of *Tat* LTR-RTs. The maximum-likelihood phylogenetic tree was reconstructed based on the amino acid sequences of the aRH domains from *Tat* representatives, as well as the RH genes of representative green plants and Archaea. The RH domains from other representative Ty3/gypsy LTR-RTs and vertebrate retroviruses, as well as representative RH genes from fungi and animals were used to root the aRH cluster (not shown). Tat LTR-RTs structures (colored circles), clusters (A-L, Z1-Z2), taxonomy, and element names are as in Figure 2, Supplementary Table S1 and Table S3. aLRT/UFboot branch support values are shown only for major selected clusters/nodes.

Table S1

Diversity, taxonomic position, and data sources of the studied plant genomes

Taxonomy			Source Database	Assembly version	Identifier in Source Database	Last accessed
Phylum/Clade (common name)	Class/order	Species				
Prasinodermophyta	Prasinodermophyceae	*Prasinoderma coloniale*	CNGBdb	V2.0	CNA0013964	10.01.2023
Chlorophyta (green algae)	Mamiellophyceae	*Ostreococcus lucimarinus*	NCBI:Genome	V1.0	ASM9206v1	10.10.2019
		Ostreococcus tauri	NCBI:Genome	V2.0	version 140606	10.01.2023
		Bathycoccus prasinos	NCBI:Genome	V1.0	ASM222023v1	10.01.2023
		Micromonas pusilla	NCBI:Genome	V2.0	CCMP1545	10.01.2023
		Micromonas commoda	NCBI:Genome	V2.0	ASM9098v2	10.01.2023
	Pyramimonadophyceae	*Cymbomonas tetramitiformis*	NCBI:Genome	V1.0	ASM124769v1	10.01.2023
	Chlorophyceae	*Chlamydomonas reinhardtii*	NCBI:Genome	V5.5	Chlamydomonas_reinhardtii_v5.5	10.01.2023
		Chlamydomonas eustigma	NCBI:Genome	V1.0	C.eustigma genome v1.0	10.01.2023
		Volvox carteri	NCBI:Genome	V1.0	WGS project - ACJH01	10.01.2023
		Gonium pectorale	NCBI:Genome	V1.0	ASM158458v1	10.01.2023
		Tetrabaena socialis	NCBI:Genome	V1.0	TetSoc1	10.01.2023
		Haematococcus pluvialis	NCBI:Genome	V1.0	ASM397095v1	10.01.2023
		Yamagishiella unicocca	NCBI:Genome	V1.0	YamagishiellaPlus_1.0	10.01.2023
		Eudorina sp.	NCBI:Genome	V1.0	EudorinaFemale_1.0	10.01.2023
		Mychonastes homosphaera	NCBI:Genome	V1.0	ASM919307v1	10.01.2023
		Monoraphidium neglectum	NCBI:Genome	V1.0	mono_v1, WGS project - AYTC01	10.01.2023
		Chromochloris zofingiensis	Phytozome	v5.2.3.2	461	10.01.2023
		Tetradesmus obliquus	NCBI:Genome	V1.0	sob1, WGS project - FNXT01	10.01.2023
		Raphidocelis subcapitata	NCBI:Genome	V1.0	Rsub_1.0	10.01.2023
		Scenedesmus quadricauda	NCBI:Genome	V1.0	ASM231754v1	10.01.2023
	Ulvophyceae	*Ulva mutabilis*	NCBI:Genome	V1.0	Ulvmu_WT_fa	10.01.2023
	Trebouxiophyceae	*Parachlorella kessleri*	NCBI:Genome	V1.0	ASM1571204v1	10.01.2023
		Chlorella variabilis	NCBI:Genome	V1.0	WGS project - ADIC01	10.01.2023
		Chlorella sorokiniana	NCBI:Genome	V1.0	ASM2591765v1	10.01.2023
		Micractinium conductrix	NCBI:Genome	V2.0	ASM224581v2	10.01.2023
		Auxenochlorella protothecoides	NCBI:Genome	V1.0	ASM73321v1	10.01.2023
		Auxenochlorella pyrenoidosa	NCBI:Genome	V1.0	ASM143074v1	10.01.2023
		Helicosporidium sp.	NCBI:Genome	V1.0	Helico_v1.0	10.01.2023
		Chloroidium sp	NCBI:Genome	V1.0	ASM433562v1	10.01.2023
		Coccomyxa subellipsoidea	NCBI:Genome	V2.0	C-169	10.01.2023
		Picochlorum sp.	NCBI:Genome	V1.0	ASM87641v1	10.01.2023
		Picochlorum costavermella	NCBI:Genome	V1.0	ASM1131604v1	10.01.2023
		Botryococcus braunii	NCBI:Genome	V1.0	B_braunii_Showa_v1	10.01.2023
Streptophyta (non-Embryophyta algae)	Klebsormidiophyceae	*Klebsormidium nitens*	NCBI:Genome	V1.0	ASM70883v1	10.01.2023
	Charophyceae	*Chara braunii*	NCBI:Genome	V1.0	Cbr_1.0, WGS project - BFEA01	10.01.2023
	Chlorokybophyceae	*Chlorokybus atmophyticus*	NCBI:Genome	V1.0	ASM910322v1	10.10.2020
	Mesostigmatophyceae	*Mesostigma viride*	NCBI:Genome	V1.0	ASM974604v1	10.01.2023
	Zygnemophyceae	*Mesotaenium endlicherianum*	NCBI:Genome	V1.0	ASM960273v1	10.01.2023
		Spirogloea muscicola	NCBI:Genome	V1.0	ASM960272v1	10.01.2023
Marchantiophyta (liverworts)	Marchantiopsida	*Marchantia polymorpha*	NCBI:Genome	V1.0	Marchanta_polymorpha_v1	10.01.2023
		Marchantia inflexa	NCBI:Genome	V1.0	M_inflexa_v1.1	10.01.2023
Anthocerotophyta (hornworts)	Anthocerotopsida	*Anthoceros angustus*	NCBI:Genome	V1.0	ASM1090916v1	10.01.2023
		Anthoceros punctatus	uzh.ch	Draft	A. agrestis [oxford]	10.01.2023
		Anthoceros agrestis	uzh.ch	Draft	A. punctatus	10.01.2023
Bryophyta (mosses)	Sphagnopsida	*Sphagnum magellanicum*	Phytozome	V1.1	521	10.01.2023
		Sphagnum fallax	Phytozome	V1.1	522	10.01.2023
	Bryopsida	*Physcomitrella patens*	NCBI:Genome	V3.0	Phypa V3	10.01.2023
		Pleurozium schreberi	NCBI:Genome	V1.0	SU_Pschr_1.0	10.01.2023
		Calohypnum plumiforme	GSA	V1.0	CRX071737	10.01.2023
		Ceratodon purpureus	Phytozome	V1.1	R40 v1.1	10.01.2023
		Fontinalis antipyretica	CNGBdb	V2.1.1	CNA0007312	10.01.2023
		Syntrichia caninervis	NCBI:Genome	V2.0	UMC_Scan_2.0	10.01.2023
Lycopodiophyta (lycophytes)	Lycopodiopsida	*Selaginella tamariscina*	NCBI:Genome	V1.0	ASM302478v1	10.01.2023
		Selaginella moellendorffii	NCBI:Genome	V1.0	WGS project - ADFJ01	10.01.2023
		Selaginella kraussiana	NCBI:Genome	V1.0	ASM102113v1	10.01.2023
		Isoetes engelmannii	NCBI:Genome	V1.0	ASM1176348v1	10.01.2023
Polypodiophyta (ferns)	Polypodiopsida	*Polypodium sp*	digitalcommons. usu. edu/fern_genome /2	Draft	Polypodium_clc_k31	13.01.2020
		Pteridium sp		Draft	Pteridium_clc_hybrid	10.01.2023
		Plagiogyria sp		Draft	Plagiogyria_clc_k31	10.01.2023
		Dipteris sp		Draft	Dipteris_clc_k31	10.01.2023
		Cystopteris sp		Draft	Cystopteris_clc_k31	10.01.2023

		Ceratopteris sp		Draft	ceratopteris_clc_k31.	10.01.2023
		Ceratopteris richardii	NCBI:Genome	V1.1	CFernv1.1	10.01.2023
		Salvinia cucullata	Fernbase	V1.2	Salvinia_asm_v1.2	10.01.2023
		Azolla filiculoides	Fernbase	V1.1	Azolla_asm_v1.1	10.01.2023
Gnetophyta	Gnetopsida	*Gnetum montanum*	DRYAD	'final'	dryad.ht76hdrdr	10.01.2023
		Welwitschia mirabilis	DRYAD	'final'	dryad.ht76hdrdr	10.01.2023
Ginkgophyta	Ginkgoopsida	*Ginkgo biloba*	GigaDB	V1.0	TM011 (non-HiC version)	10.01.2023
Cycadophyta	Cycadopsida	*Cycas panzhihuaensis*	NCBI:Genome	V1.0	ASM2321339v1	10.01.2023
Pinopsida (gymnosperms)	Pinopsida (conifers I clade - order Pinales)	*Pseudotsuga menziesii*	NCBI:Genome	V1.0	DougFir1.0	10.01.2023
		Abies alba	TreeGenesDB	V1.1	Abal.1_1.fa	10.01.2023
		Pinus taeda	NCBI:Genome	V2.0	Ptaeda2.0	10.01.2023
		Pinus lambertiana	NCBI:Genome	V1.0	WGS project - LMTP01	10.01.2023
		Picea abies	NCBI:Genome	V1.0	Pabies01	10.01.2023
		Larix sibirica	NCBI:Genome	V0.1	LarixSibirica0.1	10.01.2023
		Picea sitchensis	NCBI:Genome	V1.0	Q903_v1	10.01.2023
		Larix kaempferi	NCBI:Genome	V1.0	ASM1317126v1	10.01.2023
		Picea glauca	NCBI:Genome	V5.0	PG29_v5	10.01.2023
	Pinopsida (conifers II clade - order Cupressales)	*Sequoiadendron giganteum*	NCBI:Genome	V2.0	SEGI.2.0	10.01.2023
		Sequoia sempervirens	NCBI:Genome	V1.0	SESE.1.0	10.01.2023
		Thuja plicata	Phytozome	v3.1	572	10.01.2023
		Taxus wallichiana	NCBI:Genome	V1.0	ASM1834077v1	10.01.2023
Magnoliophyta (angiosperms, flowering plants)	Magnoliopsida	*Amborella trichopoda*	NCBI:Genome	V1.0	AMTR1.0	10.01.2023
		Nymphaea colorata	NCBI:Genome	V1.0	Nym, WGS project - CABVML01	10.01.2023
		Nymphaea thermarum	NCBI:Genome	V1.0	ASM1179976v1	10.01.2023
		Aristolochia fimbriata	NCBI:Genome	V1.0	ASM1984555v1	10.01.2023
		Liriodendron chinense	NCBI:Genome	V2.0	NJFU_Lchi_2.0	10.01.2023
		Chimonanthus salicifolius	NCBI:Genome	V1.0	ASM1335033v1	10.01.2023
		Cinnamomum kanehirae	NCBI:Genome	V1.0	ASBRC_Ckan_1.0	10.01.2023
		Persea americana	NCBI:Genome	V1.0	Hass1.0, WGS project - NXHZ01	10.01.2023
		Litsea cubeba	NCBI:Genome	V1.0	ASM1293172v1	10.01.2023

Table S2

Summary of the identified *Tat* LTR retrotransposons' (LTR-RTs) diversity†

Species	Number of Tat LTR-RTs without aRH (Tat Z)	Number of LTR-RTs with aRH, N	Structure ID, # and its fraction among others (%)*	Subclustered lineages, N	Elements with detected LTRs, %	Mean LTR identity, %	Maximum LTR identity, %	Elements with 100% identical LTRs, N
M. polymorpha	178	0	-	-	-	-	-	-
M. inflexa	148	0	-	-	-	-	-	-
A. angustus	11	4	#1 (100)	Singlets	25.00	97.1	97.1	0
A. punctatus	32	9	#1 (66.7)	Singlets	33.33	91.7	97.7	0
			#5 (33.3)	Singlets	33.33	91.3	91.3	0
A. agrestis	72	19	#1 (84.2)	2	50.0	99.7	99.7	0
			#5 (15.8)	Singlets	66.67	96.5	96.9	0
S. magellanicum	49	0	-	-	-	-	-	-
S. fallax	53	0	-	-	-	-	-	-
P. patens	783	0	-	-	-	-	-	-
P. schreberi	9	7	#4 (100)	Singlets	42.85	98.5	96.7	0
C. plumiforme	24	8	#4 (100)	Singlets	87.50	93.9	92.8	0
C. purpureus	952	5	#4 (100)	1	25.0	99.7	100.0	1
F. antipyretica	381	0	-	-	-	-	-	-
S. caninervis	119	12	#4 (91.7)	1	33.3	90.7	95.3	0
S. tamariscina	303	73	#1 (20.8)	1	100.0	98.6	99.2	0
			#4 (70.8)	3	60.0	95.8	99.4	0
S. moellendorffii	82	51	#2 (100)	2	40.7	93.1	99.5	0
S. kraussiana	3	2	#2 (50.0)	Singlets	0	-	-	-
			#3 (50.0)	Singlets	0	-	-	-
I. engelmannii	116	110	#2 (88.1)	3	35.7	-	96.7	0
Polypodium sp	0	0	-	-	-	-	-	-
Pteridium sp	9	3	#1 (100)	Singlets	33.33	-	-	-
Plagiogyria sp	8	9	#1 (100)	1	50.0	-	-	-
Dipteris sp	5	0	-	-	-	-	-	-
Cystopteris sp	5	4	#1 (100)	Singlets	0	-	-	-
Ceratopteris sp	0	0	-	-	-	-	-	-
C. richardii	3283	53	#1 (100)	2	30.0	90.9	91.1	0
S. cucullata	46	17	#1 (100)	1	0.0	91.9	94.3	0
A. filiculoides	2436	167	#1 (100)	2	30.4	98.5	100.0	23
G. montanum	3	26150	#1 (88.7)	58	1.6	88.9	100.0	3
			#2 (11.1)	17	11.6	92.4	98.6	0
W. mirabilis	21	879	#1 (90.0)	15	46.7	88.1	98.2	0
			#2 (0.6)	Singlets	0	-	-	-
G. biloba	19833	21357	#1 (23.5)	22	54.7	92.0	99.2	0
			#2 (71.5)	26	15.2	88.8	100.0	1
			#3 (3.8)	10	79.8	89.3	97.6	0
C. panzhihuaensis	29603	2906	#2 (10.7)	7	30.6	82.1	87.1	0
			#3 (68.0)	10	72.4	89.8	98.5	0
P. menziesii	37432	65496	#2 (66.2)	61	18.6	93.2	100.0	2
			#3 (31.6)	40	80.4	92.1	100.0	1
A. alba	42997	86276	#2 (73.2)	148	20.4	90.9	100.0	13
			#3 (25.2)	75	73.3	91.4	98.7	0
P. taeda	24906	39538	#2 (78.5)	64	29.9	91.3	100.0	13
			#3 (18.7)	30	72.0	91.7	100.0	2
P. lambertiana	24900	49880	#2 (82.9)	160	20.5	91.6	100.0	3
			#3 (14.9)	34	70.0	91.1	98.2	0
P. abies	11967	29708	#2 (86.3)	37	57.4	90.6	100.0	2
			#3 (12.5)	12	80.5	88.9	98.1	0
L. sibirica	170	2135	#2 (93.2)	13	30.8	88.0	100.0	1
			#3 (1.6)	3	25.0	-	-	-
P. sitchensis	29162	50267	#2 (85.7)	63	41.7	90.1	100.0	23
			#3 (13.5)	14	65.9	89.4	95.1	0
L. kaempferi	31175	27181	#2 (50.2)	50	22.9	92.7	100.0	5
			#3 (47.2)	61	59.3	92.5	100.0	2
P. glauca	6744	21831	#2 (80.3)	36	28.2	91.5	100.0	68
			#3 (17.9)	14	68.1	93.5	100.0	14
S. giganteum	169	56032	#1 (65.0)	109	67.1	90.2	100.0	125
			#2 (34.7)	36	34.5	90.5	100.0	54
S. sempervirens	2409	164834	#1 (59.3)	334	69.5	91.4	100.0	66
			#2 (40.2)	106	38.2	92.8	100.0	25
T. plicata	1769	49262	#1 (63.2)	79	67.4	91.9	100.0	12

			#2 (36.3)	40	31.7	92.1	100.0	13
T. wallichiana	25761	73178	#1 (34.3)	281	54.4	92.0	100.0	26
			#2 (64.7)	294	3.4	91.3	99.7	0
A. trichopoda	0	1702	#1 (99.8)	63	30.5	81.4	91.5	0
N. colorata	0	314	#1 (100)	7	26.9	95.2	100.0	1
N. thermarum	0	160	#1 (100)	5	19.0	94.8	99.0	0
A. fimbriata	0	1322	#1 (99.9)	14	72.1	93.1	100.0	1
L. chinense	0	13029	#1 (99.6)	140	89.8	90.0	100.0	61
C. salicifolius	0	1	#1 (100)	Singlets	0	-	-	-
C. kanehirae	0	1624	#1 (99.0)	36	62.3	93.1	100.0	7
P. americana	0	2862	#1 (99.5)	39	61.5	94.3	100.0	3
L. cubeba	0	10800	#1 (99.4)	39	29.5	97.8	100.0	153

† species taxonomy as in Table S1.

* the total of frequencies may not match 100% due to chimeric elements with unassigned structures.

Table S3

Summary of the identified *Tat* LTR-RTs representatives

Name at trees	Species	Structure	Cluster	LTR_identity, %	LTR_length, nt	Structural annotation
Aagr_2	*A. agrestis*	#1	A	99.734	376	GAG.PRo.gRT.gRH.aRH.INT
Aang_2	*A. angustus*	#1	A	97.143	455	PRo.gRT.gRH.aRH.INT
Apun_2	*A. punctatus*	#1	A	97.657	683	GAG.PRo.gRT.gRH.aRH.INT
Aagr_3	*A. agrestis*	#5	A	96.884	674	GAG.PRo.gRT.gRH.aRH.gRH.INT
Apun_3	*A. punctatus*	#5	A	91.311	679	GAG.PRo.gRT.gRH.aRH.gRH.INT
Aagr_4	*A. agrestis*	#5	A	96.041	682	GAG.PRo.gRT.gRH.aRH.gRH.INT
Aagr_5	*A. agrestis*	#5	A	-	-	gRT.gRH.aRH.gRH.INT
Apun_4	*A. punctatus*	#5	A	-	-	GAG.PRo.gRT.gRH.aRH.gRH.INT
Apun_5	*A. punctatus*	#5	A	-	-	PRo.gRT.gRH.aRH.gRH
Cplu_2	*C. plumiforme*	#4	B	93.93	313	aRH.GAG.PRo.gRT.gRH.INT
Cpur_2	*C. purpureus*	#4	B	99.157	356	aRH.GAG.PRo.gRT.gRH.INT
Psch_2	*P. schreberi*	#4	B	94.688	320	aRH.GAG.PRo.gRT.gRH.INT
Scan_2	*S. caninervis*	#4	B	95.312	256	aRH.GAG.PRo.gRT.gRH.INT
Stam_2	*S. tamariscina*	#1	C	97.468	237	GAG.PRo.gRT.gRH.aRH.INT
Stam_3	*S. tamariscina*	#4	C	96.134	776	aRH.GAG.PRo.gRT.gRH.INT
Afil_2	*A. filiculoides*	#1	D	99.844	641	GAG.PRo.gRT.gRH.aRH.INT
Cric_2	*C. richardii*	#1	D	-	-	GAG.PRo.gRT.gRH.aRH.INT
Cysto_sp_2	*Cystopteris sp*	#1	D	-	-	gRT.gRH.aRH.INT
Plagi_sp_2	*Plagiogyria sp*	#1	D	-	-	PRo.gRT.gRH.aRH.INT
Pteri_sp_1	*Pteridium sp*	#1	D	-	-	GAG.PRo.gRT.gRH.aRH.INT
Scuc_2	*S. cucullata*	#1	D	93.557	714	GAG.PRo.gRT.gRH.aRH.INT
Aalb_2	*A. alba*	#2	E	90.582	361	PRo.aRH.gRT.gRH.INT
Cpan_2	*C. panzhihuaensis*	#2	E	-	-	PRo.aRH.gRT.gRH.INT
Gbil_3	*G. biloba*	#2	E	91.667	1068	GAG.PRo.aRH.gRT.gRH.INT
Gmon_2	*G. montanum*	#2	E	98.578	1125	GAG.PRo.aRH.gRT.gRH.INT
Lkae_2	*L. kaempferi*	#2	E	99.917	1212	GAG.PRo.aRH.gRT.gRH.INT
Lsib_2	*L. sibirica*	#2	E	-	-	GAG.PRo.aRH.gRT.gRH.INT
Pabi_2	*P. abies*	#2	E	98.609	791	GAG.PRo.aRH.gRT.gRH.INT
Pgla_2	*P. glauca*	#2	E	96.273	483	GAG.PRo.aRH.gRT.gRH.INT
Plam_2	*P. lambertiana*	#2	E	96.42	810	PRo.aRH.gRT.gRH.INT
Pmen_2	*P. menziesii*	#2	E	98.441	3207	GAG.PRo.aRH.gRT.gRH.INT
Psit_2	*P. sitchensis*	#2	E	86.634	1414	GAG.PRo.aRH.gRT.gRH.INT
Ptae_2	*P. taeda*	#2	E	99.244	1323	GAG.PRo.aRH.gRT.gRH.INT
Sgig_3	*S. giganteum*	#2	E	99.27	137	GAG.PRo.aRH.gRT.gRH.INT
Ssem_3	*S. sempervirens*	#2	E	96.359	1950	GAG.PRo.aRH.gRT.gRH.INT
Tpli_3	*T. plicata*	#2	E	-	-	GAG.PRo.aRH.gRT.gRH.INT
Twal_4	*T. wallichiana*	#2	E	97.205	3542	GAG.PRo.aRH.gRT.gRH.INT
Wmir_2	*W. mirabilis*	#2	E	-	-	PRo.aRH.gRT.gRH.INT
Ieng_2	*I. engelmannii*	#2	F	-	-	GAG.PRo.aRH.gRT.gRH.INT
Skra_2	*S. kraussiana*	#2	G	-	-	PRo.aRH.gRT.gRH.INT
Smoe_2	*S. moellendorffii*	#2	G	96.859	191	GAG.PRo.aRH.gRT.gRH.INT
Aalb_3	*A. alba*	#3	H	92.101	1114	gRT.gRH.INT.aRH
Lkae_3	*L. kaempferi*	#3	H	96.393	1691	PRo.gRT.gRH.INT.aRH
Lsib_3	*L. sibirica*	#3	H	-	-	GAG.PRo.gRT.gRH.INT.aRH
Pabi_3	*P. abies*	#3	H	-	-	GAG.PRo.gRT.gRH.INT.aRH
Pgla_3	*P. glauca*	#3	H	95.148	1484	PRo.gRT.gRH.INT.aRH
Plam_3	*P. lambertiana*	#3	H	90.803	598	GAG.PRo.gRT.gRH.INT.aRH
Pmen_3	*P. menziesii*	#3	H	91.066	985	PRo.gRT.gRH.INT.aRH
Psit_3	*P. sitchensis*	#3	H	93.917	1759	GAG.PRo.gRT.gRH.INT.aRH
Ptae_3	*P. taeda*	#3	H	98.157	217	GAG.PRo.gRT.gRH.INT.aRH
Cpan_3	*C. panzhihuaensis*	#3	I	91.635	263	GAG.PRo.gRT.gRH.INT.aRH
Gbil_4	*G. biloba*	#3	I	92.795	347	GAG.PRo.gRT.gRH.INT.aRH
Gbil_5	*G. biloba*	#1	J	97.572	659	gRT.gRH.aRH.INT
Sgig_2	*S. giganteum*	#1	J	98.66	1717	gRT.gRH.aRH.INT

Ssem_2	*S. sempervirens*	#1	J	98.718	156	gRT.gRH.aRH.INT
Tpli_2	*T. plicata*	#1	J	98.193	166	gRT.gRH.aRH.INT
Twal_3	*T. wallichiana*	#1	J	91.176	102	gRT.gRH.aRH.INT
Twal_5	*T. wallichiana*	#2	J	100.0	129	GAG.PRo.aRH.gRT.gRH.INT
Gmon_1	*G. montanum*	#1	K	96.486	626	gRT.gRH.aRH.INT
Wmir_1	*W. mirabilis*	#1	K	88.063	1977	PRo.gRT.gRH.aRH.INT
Afim_1	*A. fimbriata*	#1	L	94.969	159	gRT.gRH.aRH.INT
Atri_1	*A. trichopoda*	#1	L	91.489	141	GAG.gRT.gRH.aRH.INT
Ckan_1	*C. kanehirae*	#1	L	100.0	570	GAG.PRo.gRT.gRH.aRH.INT
Csal_1	*C. salicifolius*	#1	L	-	-	gRT.gRH.aRH.INT
Lchi_1	*L. chinense*	#1	L	90.957	752	GAG.PRo.gRT.gRH.aRH.INT
Lcub_1	*L. cubeba*	#1	L	98.917	739	GAG.PRo.gRT.gRH.aRH.INT
Ncol_1	*N. colorata*	#1	L	97.389	651	GAG.PRo.gRT.gRH.aRH.INT
Nthe_1	*N. thermarum*	#1	L	98.417	379	GAG.PRo.gRT.gRH.aRH.INT
Pame_1	*P. americana*	#1	L	86.624	157	GAG.PRo.gRT.gRH.aRH.INT
Aagr_1	*A. agrestis*	no aRH	Z1	100.0	356	GAG.PRo.gRT.gRH.INT
Aalb_1	*A. alba*	no aRH	Z1	94.231	312	PRo.gRT.gRH.INT
Aang_1	*A. angustus*	no aRH	Z1	82.21	1484	gRT.gRH.INT
Afil_1	*A. filiculoides*	no aRH	Z1	99.728	368	GAG.PRo.gRT.gRH.INT
Apun_1	*A. punctatus*	no aRH	Z1	94.866	409	GAG.PRo.gRT.gRH.INT
Cpan_1	*C. panzhihuaensis*	no aRH	Z1	89.357	855	PRo.GAG.gRT.gRH.INT
Cplu_1	*C. plumiforme*	no aRH	Z1	99.794	973	GAG.PRo.gRT.gRH.INT
Cpur_1	*C. purpureus*	no aRH	Z1	100.0	423	GAG.PRo.gRT.gRH.INT
Cric_1	*C. richardii*	no aRH	Z1	-	-	GAG.PRo.gRT.gRH.INT
Cysto_sp_1	*Cystopteris sp*	no aRH	Z1	-	-	PRo.gRT.gRH.INT
Dipte_sp_1	*Dipteris sp*	no aRH	Z1	-	-	GAG.PRo.gRT.gRH.INT
Fant_1	*F. antipyretica*	no aRH	Z1	99.132	461	PRo.gRT.gRH.INT
Gbil_1	*G. biloba*	no aRH	Z1	-	-	PRo.gRT.gRH.INT
Ieng_1	*I. engelmannii*	no aRH	Z1	97.949	585	GAG.PRo.gRT.gRH.INT
Lkae_1	*L. kaempferi*	no aRH	Z1	98.221	1743	GAG.PRo.gRT.gRH.INT
Lsib_1	*L. sibirica*	no aRH	Z1	99.942	1725	GAG.PRo.gRT.gRH.INT
Minf_1	*M. inflexa*	no aRH	Z1	-	-	PRo.gRT.gRH.INT
Mpol_1	*M. polymorpha*	no aRH	Z1	96.445	2672	GAG.PRo.gRT.gRH.INT
Pabi_1	*P. abies*	no aRH	Z1	90.094	848	GAG.PRo.gRT.gRH.INT
Pgla_1	*P. glauca*	no aRH	Z1	100.0	851	PRo.gRT.gRH.INT
Plagi_sp_1	*Plagiogyria sp*	no aRH	Z1	-	-	GAG.PRo.gRT.gRH.INT
Plam_1	*P. lambertiana*	no aRH	Z1	84.736	832	GAG.PRo.gRT.gRH.INT
Pmen_1	*P. menziesii*	no aRH	Z1	96.288	943	gRT.gRH.INT
Ppat_1	*P. patens*	no aRH	Z1	90.043	703	GAG.PRo.gRT.gRH.INT
Psch_1	*P. schreberi*	no aRH	Z1	94.057	1161	GAG.PRo.gRT.gRH.INT
Psit_1	*P. sitchensis*	no aRH	Z1	91.453	351	GAG.PRo.gRT.gRH.INT
Ptae_1	*P. taeda*	no aRH	Z1	86.371	587	PRo.gRT.gRH.INT
Scan_1	*S. caninervis*	no aRH	Z1	100.0	108	GAG.PRo.gRT.gRH.INT
Scuc_1	*S. cucullata*	no aRH	Z1	81.143	175	GAG.PRo.gRT.gRH.INT
Sfal_1	*S. fallax*	no aRH	Z1	95.918	294	GAG.PRo.gRT.gRH.INT
Sgig_1	*S. giganteum*	no aRH	Z1	88.91	523	GAG.PRo.gRT.gRH.INT
Skra_1	*S. kraussiana*	no aRH	Z1	-	-	GAG.PRo.gRT.gRH.INT
Smag_1	*S. magellanicum*	no aRH	Z1	-	-	GAG.PRo.gRT.gRH.INT
Smoe_1	*S. moellendorffii*	no aRH	Z1	99.698	331	GAG.PRo.gRT.gRH.INT
Ssem_1	*S. sempervirens*	no aRH	Z1	100.0	395	GAG.PRo.gRT.gRH.INT
Stam_1	*S. tamariscina*	no aRH	Z1	96.147	545	GAG.PRo.gRT.gRH.INT
Tpli_1	*T. plicata*	no aRH	Z1	98.235	340	PRo.gRT.gRH.INT
Twal_1	*T. wallichiana*	no aRH	Z1	84.469	631	PRo.gRT.gRH.INT
Mpol_2	*M. polymorpha*	no aRH	Z2	86.111	612	GAG.PRo.gRT.gRH.INT
Sfal_2	*S. fallax*	no aRH	Z2	98.387	310	GAG.PRo.gRT.gRH.INT
Smag_2	*S. magellanicum*	no aRH	Z2	94.245	139	GAG.PRo.gRT.gRH.INT
Twal_2	*T. wallichiana*	no aRH	Z2	96.016	1456	GAG.PRo.gRT.gRH.INT
Gbil_2	*G. biloba*	no aRH	Z2	82.94	381	PRo.gRT.gRH.INT
